# The Prognostic Assessment of CDC20 in Patients with Renal Clear Cell Carcinoma and Its Relationship with Body Immunity

**DOI:** 10.1155/2022/7727539

**Published:** 2022-06-08

**Authors:** Jiaqi Shi, Yinhao Chen, Xiameng Gu, Xuerong Wang, Jing Liu, Xiaolan Chen

**Affiliations:** ^1^Department of Nephrology, Affiliated Hospital of Nantong University, Nantong 226001, Jiangsu, China; ^2^Nantong University School of Medicine, Nantong 226001, Jiangsu, China; ^3^Department of Urology, Affiliated Hospital of Nantong University, Nantong 226001, Jiangsu, China

## Abstract

This article analyzes the relationship between cell division cycle (CDC20) molecules and oncology outcomes in patients with renal clear cell carcinoma (KIRC). CDC20 appears to act as a regulatory protein interacting with many other proteins at multiple points in the cycle. The RNA sequencing data and corresponding clinical information of CDC20 molecules were obtained from The Cancer Genome Atlas (TCGA) database. The expression of CDC20 in kidney renal clear cell carcinoma tissue and adjacent normal tissue was detected by immunohistochemical methods. Logistic analysis was performed to analyze the role of CDC20 in the clinicopathological characteristics and prognosis of KIRC. Gene Set Enrichment Analysis (GSEA) was used to identify the signal pathways which were related to CDC20. Independent prognostic factors were evaluated using univariate and multivariate Cox regression analysis. A nomogram involved in CDC20 expression and clinicopathological variables was conducted to predict overall survival (OS) in KIRC patients at 1, 3, and 5 years. Furthermore, the relation between CDC20 and immunity was also studied. Our results showed that CDC20 was upregulated in kidney renal clear cell carcinoma tissues, accompanying shorter OS (all *P* < 0.05). According to the results obtained by immunohistochemistry and TCGA database, CDC20 was significantly upregulated in kidney renal clear cell carcinoma tissues compared with neighboring normal kidney tissues. Univariate and multivariate Cox regression analysis showed that high expression of CDC20 was an independent prognostic factor of poor prognosis in kidney renal clear cell carcinoma patients (all *P* < 0.05). GSEA analysis suggested that the high expression of CDC20 was related to eight multiple signaling pathways. In addition, CDC20 was linked to tumour mutation burden (TMB), immune checkpoint molecules, tumour microenvironment, and immunological infiltration.

## 1. Introduction

Solid kidney tumors (SKT) are one of the most common malignant tumors globally, accounting for 2%-3% of adult malignancies [1, 2]. KIRC is one of the most aggressive malignancies in the urinary system. According to statistics, its incidence accounts for 80% of tumors that have become the focus of treatment and translational research. In most cases, KIRC is not sensitive to radiotherapy and chemotherapy and can only be removed by surgery. However, although surgeries have been operated on the KIRC patients, 30% of patients develop distant metastases finally [1, 3]. With the development of science and technology, the OS of KIRC patients has been improved by introducing immunotherapy, and established angiogenesis therapies have also been combined in selected patients. However, the treatment response of patients is different [4]. Therefore, for targeted therapy of KIRC patients, the discovery of predictive biomarkers is essential. After CDC20 was discovered nearly half a century ago, its initial role was mainly elucidated in regulating the progression of the cell cycle. Cells with CDC20 mutations prevent cell division and prevent the cell cycle process to later stages and chromosome separation [5]. As we all know, CDC20 (cell division cycle 20 homology) can activate APC (late promotion complex), which plays a key role in regulating the cell cycle progression in M and G1 phases [6]. In terms of mechanism, CDC20 targets a variety of substrates, including p21 [7], Nek2A (NIMA-related kinase 2) [8, 9], cyclin A [10, 11], SMAR1 (scaffold matrix junction region binding protein 1) [12], and Mcl-1 (myeloid leukemia-1) [13] to control the cell cycle process. It has recently been reported that the abnormal expression of CDC20 is associated with the malignant progression and poor prognosis of urothelial bladder cancer, pancreatic cancer, gastric cancer, astrocytoma, lung adenocarcinoma, hepatocellular carcinoma, and oral squamous cell carcinoma [14]. In addition, the high expression of CDC20 is significantly related to the advanced tumor stages of breast cancer [15], prostate cancer [16], colon cancer, and endometrial cancer [14]. Therefore, CDC20 may become a promising therapeutic target in the fight against human cancer. More and more evidences show that CDC20 acts as an oncoprotein in tumorigenesis [6]. Therefore, we examined the expression of CDC20 in renal cell carcinoma and clinicopathological characteristics and evaluated the prognostic value of CDC20 in renal cell carcinoma. In addition, the related signal pathways and their relationship with immunity were analyzed, in order to provide reference for future research.

The paper is organized in the following way: Section 1 presents the introduction.  Section 2 describes the materials and methods, and a few subsections describe the experiment.  Section 3 discusses the paper results which are used in the experiment analysis.  Section 4 defines the discussion of all experiments and results.  Section 5 concludes the article.

## 2. Materials and Methods

This section defines the data acquisition and processing for KIRC patients and then describes many experiments.

### 2.1. Data Acquisition and Processing

The gene transcriptome and related clinical data of KIRC patients were downloaded from the TCGA database, including 72 normal kidney tissue samples and 539 KIRC samples. Then, we excluded cases that lacked key clinical information and applied R (https://www.r-project.org/) software to further analyze the genetic profiles and corresponding clinical information of the remaining tissue samples. The software packages Limma and Beeswarm were used to analyze the different expression levels of CDC20 mRNA in KIRC patients in the TCGA database. Finally, the cut-off standard of statistically significant genes was set as |log2 times change (FC) >2 and the corresponding *P* < 0.05. All data were downloaded by the type of Fragments per Kilobase Million (FPKM). At the same time, we also verified the expression of CDC20 in the GSE 15641 dataset, including 23 normal kidney tissue samples and 32 KIRC samples. All data were downloaded from the oncoming website in the form of log2 median-centered intensity.

### 2.2. Immunohistochemical Staining

From 2019 to 2020, the KIRC tissues and corresponding normal adjacent tissues of 10 patients undergoing radical renal resection in the Affiliated Hospital of Nantong University were collected for immunohistochemical staining and data analysis. In order to further verify the expression of the molecule in the tissues, the 10 pairs of kidney cancer tissues (KCT) and their corresponding adjacent normal tissues were fixed with formalin, embedded in paraffin, and subjected to the next step of CDC20 immunohistochemistry (IHC) staining. The CDC20 antibody for immunohistochemical staining was from Proteintech (about 10252-1-AP). After taking paraffin, hydrating, and blocking, add anti-CDC20 goat polyclonal antibody (diluted at 1 : 100) and incubate overnight at 4°C. Finally, under the microscope, all the sections were evaluated by comparing the staining of each kidney cancer specimen with adjacent specimens. The scores were mainly based on the number of positive cells and the staining intensity. Dyeing intensity score 0∼3, respectively, represents no dyeing to brown. Positive cell score: 0, <5%; 1, 6–25%; 2, average 26%–75%; 3, >75%. Then, calculate the product of the IHC total score, which is divided into 4 levels: 0, negative; 1∼4, weakly positive (+); 5∼8, positive (+); 9∼12, strong positive (+).

### 2.3. Functional Pathway Enrichment in KIRC by TCGA analysis

To investigate the gene expression of tumor and adjacent nontumor tissue in the TCGA database, we used Limma package (https://www.bioconductor.org/packages/release/bioc/html/limma.html), DEGs between tumor tissue and paracarcinoma tissues in the TCGA database were investigated, and the CDC20 expression in different cancer clinical stages was compared as well. Besides, as an encyclopedia of genes and genomes, Kyoto Encyclopedia of Genes and Genomes (KEGG) pathway enrichment analysis was conducted to seek out potential pathways, including visualization, further annotation, and integrated discovery (https://www.kegg.jp/ or https://www.genome.jp/kegg/) [17]. It was considered statistically significant as *P* value <0.05 and FDR <25%.

### 2.4. Cox Regression Analysis

We used the Cox proportional hazard regression model (significance defined as *P* < 0.05) to evaluate the expression of CDC20 on overall survival and other clinical characteristics (clinical stage, histological stage, histological grade, distant metastasis status, lymph node status, myometrial infiltration, and peritoneal cytology).

### 2.5. The Establishment of Predictive Nomogram Model

In order to screen whether CDC20 and these clinicopathological parameters can be independent factors related to OS, we established a nomogram model as planned. The R software package was used to perform both univariate and multivariate Cox hazard regression analyses to the KIRC samples from the TCGA database. In addition, in order to predict the potential OS of KIRC patients, we used the R “rms” software package and the “survival” software package to construct an effective nomogram model. After each factor was divided into points, we added the points of each parameter to calculate the total points. Finally, we verified the nomogram by using the harmonic index (c-index) and the calibration curve.

### 2.6. Relationships between CDC20 and Adjacent Genes

To assess the correlations between the CDC20 and adjacent genes, the Ensemble Genome Browser was applied to evaluate the gene expression of KIRC patients from TCGA, thus identifying these genes based on RPKM values. Then, we conducted the differential analysis of these genes. Moreover, Spearman's correlation coefficient was performed to evaluate the relationship between these adjacent genes and CDC20. All the statistical analyses were marked significantly scientific as the *P* value was <0.05.

### 2.7. Detection of Microsatellite Instability (MSI), Tumour Mutational Burden (TMB), and Neoantigens

To identify autosomal microsatellite tracts, we applied MISA (Microsatellite, a marking system widely used in plant genetics and forensic medicine) (https://pgrc.ipkgatersleben.de/misa/misa.html) to identify autosomal microsatellite bundles; if more than two of all five markers showed microsatellite instability, then the tumor was marked as MSI. The detailed method was shown according to the previous description [18]. Besides, we investigated the tumor mutation burden as the number of somatic nonsynonymous missense (NSM), analyzing the different expressions of transcriptome data between tumor tissue and normal tissue adjacent to cancer, as described above [19, 20]. In addition, without changing the original settings, we performed HLA detection for tumor-specific neoantigens based on the TCGA database.

### 2.8. Correlation Analysis of CDC20 in Tumour Microenvironment and Immune Infiltration

The correlation analyses aimed at the CDC20 and six immune cell infiltrations were conducted by applying the purity-adjusted Spearman to study the relationship between CDC20 and tumor microenvironment. Moreover, we evaluated KIRC patients from the following three aspects: estimated scores, stromal scores, and immune scores [21]. The estimate algorithm was implemented by using a normalized expression matrix. When the calculated *P* value was less than 0.05, the difference was statistically significant. CIBERSORT, as an important deconvolution algorithm, can predict multiple potential cell subtypes by analyzing the gene expression of the mixture [22, 23]. On the basis of a standardized database, we evaluated the cellular composition of tumor tissues to detect abundant specific cell types [21, 24]. In this study, immune cells and immune checkpoint molecules were evaluated by detecting their expression levels of them. The above analysis was performed using the free online data analysis platform Sangerbox tool (https://www.sangerbox.com/tool).

### 2.9. Statistical Analysis

R 4.0 software (https://www.r-project.org/), SPSS 24.0 (IBM, Chicago, USA), and GraphPad Prism 7.0 (San Diego, CA, USA) were applied to handle all the data. The correlation was implemented by Wilcoxon signed-rank test and logistic regression; then, the validation of predictive performance was conducted by the Kaplan–Meier curves. Also, the univariate and multivariate Cox regression analyses were conducted, respectively, to assess whether each factor could predict OS. The nomogram model was realized by using the rms package. Ultimately, we also studied CDC20 from the following three aspects: the MSI, tumor mutational burden, and neoantigens. Using an online analysis timer and other accurate analysis methods, we found the associations between CDC20 and the immune microenvironment, immune infiltrations, etc. In this study, *P* < 0.05 was considered statistically significant.

## 3. Results

### 3.1. The Expression of CDC20 in KIRC Based on TCGA

**Figure 1:**
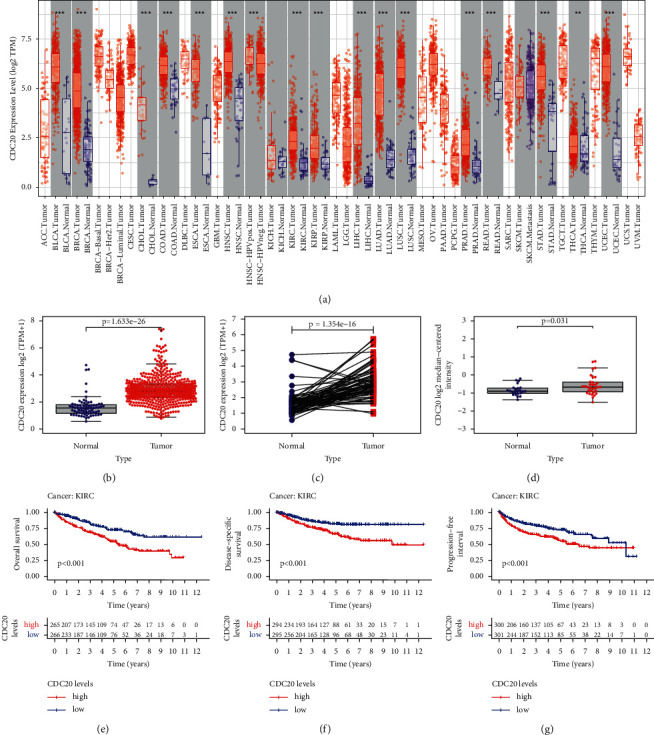
The expression of CDC20 in KIRC tissues: (a) the expression of CDC20 in different tumor tissues through the TCGA database; (b) the relative expression of CDC20 molecules in KIRC tissues and normal tissues in TCGA dataset; (c) paired box plots corresponding to the differential expression of CDC20 between KIRC and normal tissues in TCGA dataset; (d) CDC20 expression in KIRC tissue and normal kidney tissue in GSE15641 dataset; (e–g) the OS, DSS, and PFI curves of KIRC patients with high and low expression of CDC20 in the TCGA database.

The mRNA expression levels of CDC20 were acquired and investigated from TCGA database to identify the differential expression patterns between normal tissues and tumor tissues, finding that in a majority of tumor tissues, the expression of CDC20 was higher than its corresponding normal adjacent tissues, such as Bermuda Laser Class Association (BLCA), breast cancer gene (BRCA), cholangiocarcinoma (CHOL), chronic obstructive airway disease (COAD), and Emergency Services Coordinating Agency (ESCA) (*P* < 0.05). KIRC was also included in these tumors; as shown in Figure 1(a), the expression of CDC20 in renal cancer tissues was significantly higher than that of normal renal tissue (*P* < 0.001). The findings based on the TCGA database indicated that the expression of CDC20 was obviously increased in the KIRC samples compared to the normal ones (*P* < 0.001; Figure 1(b)). A pairwise boxplot was also analyzed, suggesting that tumor samples almost have a higher expression of CDC20 than normal ones (*P* < 0.001; Figure 1(c)). Moreover, we also performed the verification of CDC20 in kidney tumor tissues in the GSE15641 dataset and obtained the same results as the former (*P* < 0.05; Figure 1(d)). In addition, after classifying the KIRC patients into high- and low-risk subgroups by the expression of CDC20, the Kaplan–Meier curve was conducted, showing that patients in the low-CDC20 groups had a higher OS, DSS (disease-specific survival), and PFI (progression-free interval) than those in the high-CDC20 groups (*P* < 0.001; Figures 1(e)–1(g)).

### 3.2. Verification of the CDC20 Expression by IHC

**Figure 2:**
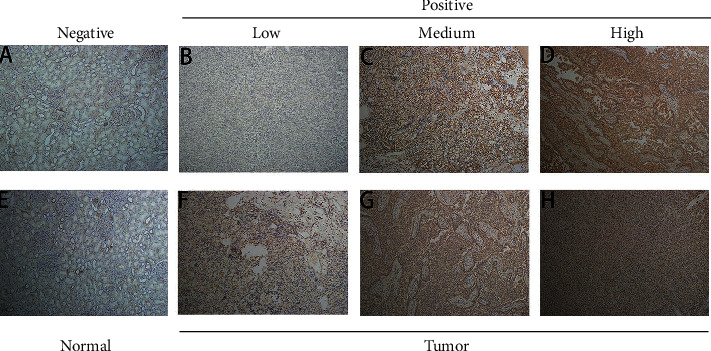
(A–H) The immunohistochemical analysis for 10 pairs of KIRC tissues and their corresponding paracancer tissues.

The results of immunohistochemical staining showed that in the 10 pairs of KIRC tissue blocks, the expression of CDC20 was higher than that of the corresponding normal tissue adjacent to cancer, and the degree of the increased CDC20 expression was individually different. According to the scoring scale, kidney tumor tissues with low, medium, and high expression of CDC20 were exemplified as shown in Figure 2 (A–H).

### 3.3. Association with the Expression of CDC20 and Clinicopathologic Variables

**Figure 3:**
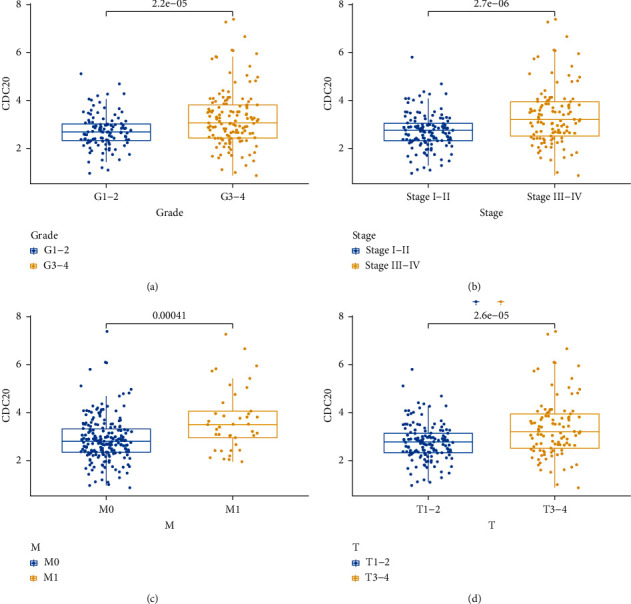
Some clinicopathological features related to the expression of CDC20: (a) grade; (b) histological stage; (c) M stage; (d) T stage.

Logistic regression analysis was performed to reveal the relationship between the expression level of CDC20 and clinicopathological variables in KIRC patients. The results in Figure 3(a) showed that the expression of CDC20 in the G3-4 stage was significantly higher than that in the G1-2 stage (*P* < 0.001); the expression of CDC20 in stage III-IV was higher than that in stage I-II (*P* < 0.001; Figure 3(b)); the expression of CDC20 in M1 stage was higher than that in M0 stage (*P*=0.00041; Figure 3(c)); the expression of CDC20 in T3-4 stage was higher than that in T1-2 stage (*P* < 0.001; Figure 3(d)). Therefore, KIRC patients with increased CDC20 expression were significantly related to histological grade, clinical stage, distant metastasis, and histological stage. The closer the KIRC was to the advanced stage, the higher the expression of CDC20 molecules.

### 3.4. CDC20 Could Serve as an Independent Risk Factor for Prognostic Evaluation of KIRC

**Figure 4:**
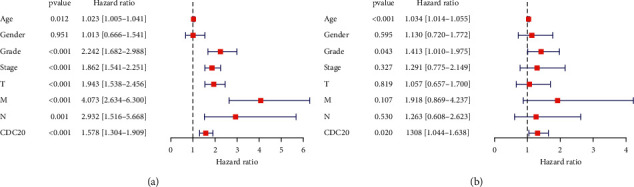
Univariate (a) and multivariate (b) Cox regression analysis of clinicopathological variables and CDC20 molecules of KIRC patients in the TCGA database.

According to the TCGA dataset, we conducted the univariate and multivariate Cox regression analyses to figure out whether CDC20 expression could serve as an independent factor associated with OS (Table 1). The results showed that, in the univariate Cox analysis, age (HR = 1.023, *P*=0.012), histological grade (HR = 2.242, *P* < 0.001), histological stage (HR = 1.862, *P* < 0.001), clinical stage (HR = 1.943, *P* < 0.001), distant metastasis (HR = 4.073, *P* < 0.001), lymph node status (HR = 2.932, *P* < 0.001), and CDC20 expression (HR = 1.578, *P* < 0.001) could serve as independent variables (Figure 4(a)). At the same time, multivariate Cox regression analysis suggested that high CDC20 expression was a categorical independent factor associated with the poor prognosis of patients with KIRC (HR = 1.308, *P*=0.020). Moreover, age (HR = 1.034, *P* < 0.001) and histological grade (HR = 1.413, *P*=0.043) were also suggested as independent variables for OS (Figure 4(b)). In summary, the results suggested that the expression of CDC20 could be used as an independent indicator of the prognosis of renal cell carcinoma, and the increased expression of CDC20 in KIRC indicated a poor prognosis.

### 3.5. Establishment of a Nomogram for Prognostic Prediction of Chronic Renal Cell Carcinoma

**Figure 5:**
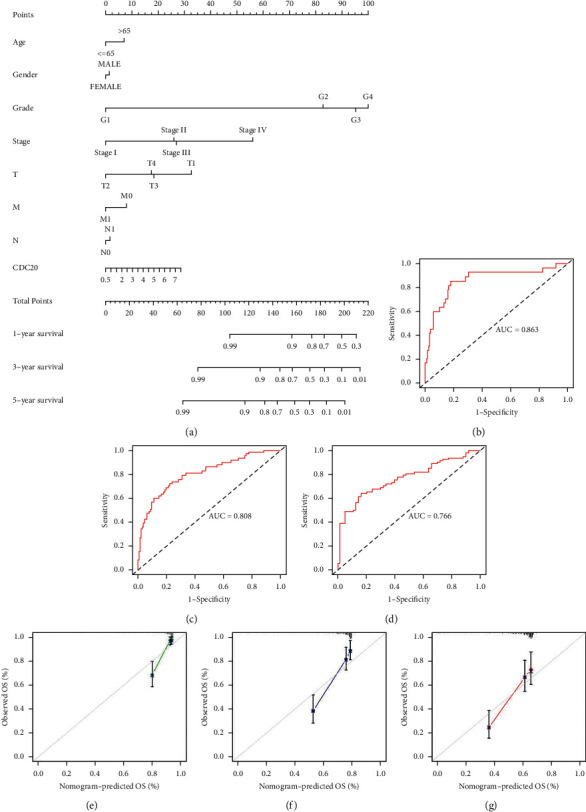
The relationship between the CDC20 molecule and the survival rate of KIRC patients verified by nomogram: (a) nomogram to predict the overall survival rate of KIRC patients in the TCGA database at 1, 3, and 5 years; (b–d) ROC curve used to identify the validity of nomogram; (e–g) calibration chart for nomogram conformance test in TCGA database.

In order to diagnose and evaluate the prognosis of renal cancer, a nomogram was constructed to analyze the relationship between 8 clinicopathological characteristics (age, gender, race, grade, stage, T, M, and N) and CDC20 gene expression (Figure 5(a)). Divide the points into various factors by the point scale in the nomogram, calculate the total number of points, and get it by calculating all the points. In addition, we could also calculate the 1-, 3-, and 5-year survival rates of patients with KIRC, and the corresponding AUC values were 0.863, 0.808, and 0.766, respectively, indicating that CDC20 could be used as an important indicator for predicting OS (Figures 5(b)–5(d); Table 2). In addition, the corresponding calibration chart provides similar prediction results as above (Figures 5(e)–5(g)), making the prediction method for KIRC patients more quantitative.

### 3.6. CDC20-Related Signaling Pathways Identified by GSEA

**Figure 6:**
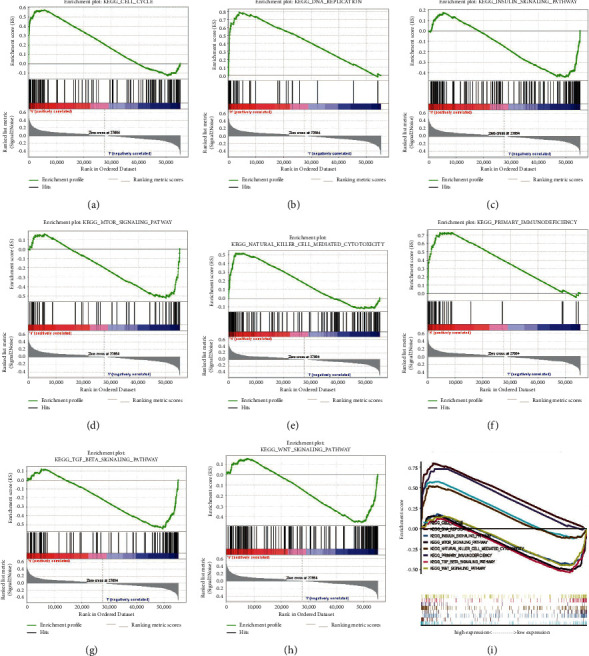
Enrichment plots by applying gene set enrichment analysis (GSEA). (a) Cell cycle; (b) DNA replication; (c) insulin signaling pathway; (d) MTOR signaling pathway; (e) natural killer cell-mediated cytotoxicity; (f) primary immunodeficiency; (g) TGF beta signaling pathway, (h) WNT signaling pathway; (i) all the eight most significantly enriched signaling pathways based on their expression.

To identify signaling pathways involved in the pathogenesis of KIRC, we conducted Gene Set Enrichment Analysis (GSEA) on datasets with different levels of CDC20 expression. According to their normalized enrichment score (NES) and false discovery rate (FDR) q value (FDR <0.05), eight signal transduction pathways were identified and selected, which were cell cycle, DNA replication, insulin signaling pathway, natural killer cell-mediated cytotoxicity, MTOR signaling pathway, primary immunodeficiency, WNT signaling pathway, and TGF beta signaling pathway (Figure 6 and Table 3). These signal transduction pathways were significantly enriched in the CDC20 expression phenotype, which might help researchers further understand the pathogenesis of KIRC.

### 3.7. Relationships between CDC20 and PPI, MSI, TMB, and Neoantigen in KIRC

**Figure 7:**
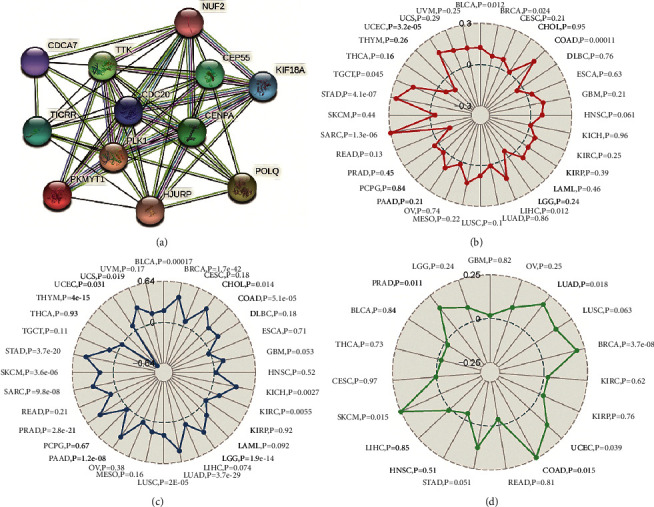
Correlations between CDC20 and PPI, TMB, MSI, and neoantigen in KIRC. (a) The CDC20 in the PPI network. (b) The relationship between CDC20 and MSI; (c) the correlation between CDC20 and TMB; (d) the correlation between CDC20 and neoantigens.

Eleven genes (NUF2, CDCA7, TTK, CEP55, KIF18A, TICRR, CENPA, PLK1, PKMYT1, HJURP, and POLQ) were meaningfully related to the expression of CDC20 according to the results analyzed by PPI network (Figure 7(a)). In addition, whether CDC20 was related to MSI, TMB, or neoantigen or not was analyzed based on the kidney renal clear cell carcinoma samples from the TCGA database. Ultimately, our results suggested that CDC20 was obviously related to TMB (*P*=0.0055). On the contrary, it had no association with neoantigen (*P*=0.62), whose data were downloaded from Sangerbox and MSI (*P*=0.25) (Figures 7(b)–7(d)).

### 3.8. Relationships between CDC20 and the Immune Infiltrations, Tumour Microenvironment, Immune Cells, and Checkpoint Molecules in KIRC

**Figure 8:**
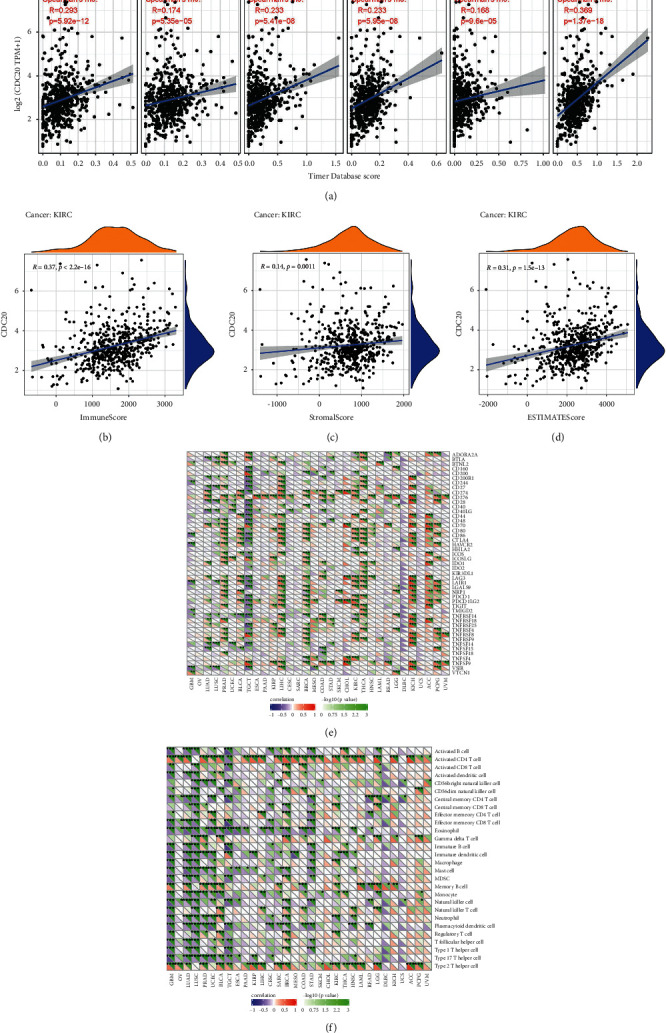
The relationship between CDC20, immune infiltration, tumor microenvironment, immune checkpoint molecules, and immune cells of KIRC. (a) The relationship between CDC20 and immune infiltration; (b–d) the relationship between CDC20 and tumor microenvironment; (e) the associations between CDC20 and immune checkpoint molecules; (f) the associations between CDC20 and immune cells

To explore the relationship between CDC20 and the 6 immune cell infiltration levels, we used the online analysis timer for correlation analysis and found that CDC20 was related with all of them, including CD4+ T cell infiltration, B cell infiltration, macrophage infiltration, neutrophil infiltration, CD8+ T cell infiltration, and dendritic cell infiltration (*P* < 0.001; Figure 8(a)). Moreover, it also showed a considerable relationship between CDC20 and immune cells (*P* < 0.001) and stromal cells (*P*=0.0011) (Figures 8(b)–8(d)); however, it had nothing to do with both of them. To analyze the correlation between CDC20 and the immune microenvironment of KIRC better, we collected more than forty common immune checkpoint molecules from Sangerbox and then analyzed the relationship between CDC20 and these immune checkpoint molecules, indicating the significant association between CDC20 and the following checkpoint molecules such as TNFSF4, TNFRSF9, TNFRSF8, TNFRSF18, and TIGIT in KIRC (Figure 8(e)). In addition, we further analyzed the correlation between CDC20 and immune cells based on the KIRC samples from the TCGA database, thus founding that CDC20 was highly bound to some immune cells, containing activated CD4 T cells, central memory CD8 T cells, eosinophils, macrophages, neutrophils etc. (Figure 8(f)).

## 4. Discussion

Among renal malignancies, renal clear cell carcinoma is one of the most common histological subtypes. Because renal clear cell carcinoma is not sensitive to radiotherapy and chemotherapy and the resistance rate of cancer tissues is high, partial or radical renal resection has become the best treatment option for patients with localized renal cancer lesions. However, about 30% of kidney cancer patients are found to have metastatic lesions at the first diagnosis and eventually develop into metastatic renal cell carcinoma (MRCC) [3, 25]. It is well known that defects in chromosome segregation may lead to aneuploidy, which is a common feature of human cancer cells and leads to genome instability [26–28]. The cell division cyclin 20 homolog (CDC20) serves as the target of the spindle assembly checkpoint (SAC) and the key cofactor of the late promotion complex or loop body (APC/C) E3 ubiquitin ligase, which regulates APC/C. The activity of ubiquitin on a specific substrate causes it to be subsequently degraded by the proteasome, thereby playing an important function in chromosome segregation and mitotic exit [29]. Therefore, we commented on the potential of CDC20 as a target for the treatment of human malignant tumors based on the bioinformatics database. As far as we know, few studies have focused on the relationship between the CDC20 gene and the prognosis of KIRC. Therefore, our research focuses on the abnormal expression of CDC20 in the kidneys of KIRC patients and its interaction with the patients' prognosis. This study analyzed the RNA sequence in KIRC from the TCGA database through bioinformatics methods. The expression level of CDC20 in tumor tissue was significantly higher than that in normal kidney tissue. We divided KIRC patients from TCGA into two groups with different expression degrees of CDC20, and Kaplan–Meier curve analyses were performed according to the two groups. The results showed that OS, DSS, and PFI of KIRC patients in the high CDC20 expression group were significantly lower than those in the low CDC20 expression group. The results of IHC showed that the expression of CDC20 in the tissues of KIRC was obviously increased compared with the normal paracancer kidney tissues. Logistic regression analysis showed that the high expression of CDC20 was related to gender, tumor grade, clinical stage, T stage, M stage, and N stage. Univariate and multivariate Cox regression analysis showed that CDC20 might be an independent prognostic indicator for patients with KIRC. In order to further study CDC20-related signal pathways, we used GSEA to analyze KIRC samples with different CDC20 expression levels from the TCGA database. Among these pathways, 8 signal pathways were closely related to the increased expression of CDC20. They were cell cycle, DNA replication, insulin signaling pathway, MTOR signaling pathway, natural killer cell-mediated cytotoxicity, primary immunodeficiency, TGF-*β* signaling pathway, and WNT signaling pathway. In terms of immunity, CDC20 was closely related to immune infiltration as well as immune cells and stromal cells, including CD4+ T cell infiltration, B cell infiltration, macrophage infiltration, neutrophil infiltration, CD8+T cell infiltration, and dendritic cell infiltration. Last but not the least, CDC20 had been confirmed to be related to immune checkpoint molecules such as TNFSF4, TNFRSF9, TNFRSF8, TNFRSF18, and TIGIT in KIRC. Evidence has shown that the expression of CDC20 was related to the metastasis and poor survival of patients with various tumors, thus playing an important role in the occurrence and development of solid tumors. In addition, similar to our results, previous studies on prostate cancer showed that CDC20 maintained the self-renewal ability of CD44+ prostate CSCs by promoting nuclear translocation and transactivation of *β*-catenin. In addition, the combination of CDC20 and CD44 or *β*-catenin could be used as an important indicator of the prognosis of prostate cancer patients [30]. There was also evidence that CDC20 molecules may activate the cell cycle of liver cancer cells, leading to a poor prognosis for liver cancer patients [31]. Moreover, previous studies had shown that dysregulation of Wnt expression could lead to a variety of developmental abnormalities and human diseases, such as congenital kidney and urinary tract abnormalities, gallbladder kidney cancer, and renal cancer, among which fructose-bisphosphate aldolase A may induce cell proliferation and metastasis through Wnt/*β*-catenin signaling pathway [32, 33]. Because the normal kidney tissue sample size in the TCGA database was small, this would cause bias in our experimental results. Our results indicated that the upregulated expression of CDC20 indicated a poor prognosis in patients with KIRC. In addition, cell cycle, DNA replication, insulin signaling pathway, MTOR signaling pathway, natural killer cell-mediated cytotoxicity, primary immunodeficiency, TGF beta signaling pathway, and WNT signaling pathway might be the main pathways regulated by CDC20. Subsequent research was expected to identify whether CDC20 could become a new target for immunotherapy. This will also provide some help for further exploration of gene-to-gene interactions. The above results all show that CDC20 plays a key role in developing KIRC. This study showed that CDC20 expression levels were elevated in patients with KIRC, and the increased expression was significantly associated with OS, immunity, and low survival. At the same time, CDC20, as an independent adverse prognostic factor, may serve as a potential prognostic marker and therapeutic target for KIRC patients, thereby helping clinicians formulate diagnosis and treatment plans, but further research is still needed to confirm.

This study has some limitations: first, selective bias cannot be avoided as a retrospective study. Second, the study object was a patient in the database. Although the sample size was large, the population diversity was poor. Third, the results of the institute were not verified by basic experiments. Finally, our study failed to explore in-depth the exact mechanisms by which CDC20 participates in KIRC, and further elucidation is needed.

## 5. Conclusion

CDC20 could be used as an independent prognostic factor of KIRC, indicating poor prognosis in patients. CDC20 could be used as an independent prognostic factor for clear cell renal cell carcinoma and was closely related to body immunity. CDC20 overexpressed in colon cancer cell lines/primary cancer tissues compared with normal colon epithelial cell lines\adjacent noncancerous tissues samples. Furthermore, CDC20 may serve as a potential prognostic biomarker of human colorectal cancer. In the future, CDC20 will be used in many other cancer therapies.

## Figures and Tables

**Table 1:** Associations with OS and clinicopathologic characteristics in KIRC patients from TCGA by applying univariate and multivariate cox analysis.

Clinical characteristics	Univariate analysis		Multivariate analysis	
	HR (95% CI)	*P* value	HR (95% CI)	*P* value
Age	1.023 (1.005–1.041)	0.012	1.034 (1.014–1.055)	0.001
Gender	1.013 (0.666–1.541)	0.951	1.130 (0.720–1.772)	0.595
Grade	2.242 (1.682–2.988)	<0.001	1.413 (1.010–1.975)	0.043
Stage	1.862 (1.541–2.251)	<0.001	1.291 (0.775–2.149)	0.327
T	1.943 (1.538–2.456)	<0.001	1.057 (0.657–1.700)	0.819
M	4.073 (2.634–6.300)	<0.001	1.918 (0.869–4.237)	0.107
N	2.932 (1.516–5.668)	0.001	1.263 (0.608–2.623)	0.530
CDC20	1.578 (1.304–1.909)	<0.001	1.308 (1.044–1.638)	0.020

**Table 2:** The AUC (area under the ROC curve) of the nomogram.

	1-year	3-year	5-year	C-index
AUC	0.863	0.808	0.766	0.757

**Table 3:** The results of gene set enrichment analysis.

MSigDB collection	Gene set name	NES	NOM *p* value
c2.cp.kegg.v7.1.symbols.gmt	CELL_CYCLE	1.908	0.017
	DNA_REPLICATION	2.099	0.002
	INSULIN_SIGNALING_PATHWAY	-1.902	0.004
	MTOR_SIGNALING_PATHWAY	-1.983	0.016
	NATURAL_KILLER_CELL_MEDIATED_CYTOTOXICITY	1.952	0.010
	PRIMARY_IMMUNODEFICIENCY	1.973	0.012
	TGF_BETA_SIGNALING_PATHWAY	-1.960	0.010
	WNT_SIGNALING_PATHWAY	-1.871	0.016

## Data Availability

The datasets used and analyzed during the current study are available from the corresponding author upon reasonable request.
